# Cognitive Bias and the Extraversion Personality Shaping the Behavior of Investors

**DOI:** 10.3389/fpsyg.2020.556506

**Published:** 2020-10-15

**Authors:** Muhammad Ishfaq, Mian Sajid Nazir, Muhammad Ali Jibran Qamar, Muhammad Usman

**Affiliations:** ^1^Department of Management Sciences, COMSATS University Islamabad, Lahore Campus, Lahore, Pakistan; ^2^Department of Management Sciences, Riphah International University, Faisalabad Campus, Faisalabad, Pakistan

**Keywords:** cognitive biases, investor behavior, personality traits, decision making, risk perception

## Abstract

The paper examines the direct and indirect effects (via investors’ risk perception) of heuristic biases on investors’ irrational behavior in decision-making. The study also investigates the moderating effect of investors’ extraversion on both the direct and the indirect associations between heuristic biases and irrational decision-making. Based on survey data collected from 247 investors registered in various brokerage houses in Pakistan and the analyses (mediation and moderation) performed using the Process Macro technique (proposed by Hayes, 2017) in SPSS, the results of this study reveal that heuristic biases positively affect investors’ irrational decision-making both directly and indirectly via risk perception. The results reveal that extraversion moderates both direct and indirect associations between heuristic biases and investors’ irrational behavior in decision-making. Our findings carry useful practical implications for organizations’ policymakers.

## Introduction

Financial theories like the efficient market hypothesis (Malkiel and Fama, 1970), the modern portfolio theory (Markowitz, 1952), and Modigliani and Miller (1958) arbitrage principle suggest that capital markets are perfectly efficient, and all investors are rational in making their investment decisions. Contrary to these theories, the prospect theory (Kahneman and Tversky, 1979) states that investors’ decisions and choices are based on their perceptions of their own utility, and they do not use all of the available information (Wang, 2017), as a result of which irrational decision-making takes place. The prospect theory also suggests that investors’ decisions are affected by potential losses and gains (Scalco et al., 2015) and that, when the option of profit and loss exists, investors prefer profit over loss (Emami et al., 2020). In other words, investors’ decisions are based on perceived gains instead of perceived losses (Kahneman and Tversky, 1979; Baker et al., 2019).

The prospect theory posits that investors’ decisions are affected by cognitive, environmental, and personal factors, so their decision-making is both bounded and irrational. Irrational investors assume that securities’ market arbitrage is imperfect, as there are no free entrances and exits, so they believe that prices cannot be in equilibrium (Baker et al., 2007). Scholarship based on the prospect theory suggests that fluctuations in stock prices are based on several factors, including human errors that arise from investors’ using instincts, feelings, habits, emotions, thinking, reason, risk, and social interactions to make decisions (Bannier and Neubert, 2016). Investors’ investment decisions involve cognitive biases (Fama, 1998; De Bondt et al., 2013) and heuristic biases (Oehler et al., 2018; Ceschi et al., 2019). Ajzen (1985) proposed a theory of planned behavior and argued that the behavior of an investor is based on cognitive biases. Investor’s attitudes move toward the behavior, subjective norms (individual thinks differently), and perceived behavioral control. Perceived behavior control refers to the investor’s belief that they can control any situation or behavior. The theory of planned behavior refers to individual beliefs and behavior. Moreover, perceived behavioral control is linked with the two magnitudes: self-efficacy and controllability. Self-efficacy shows that an investor can face or bear the difficulty, or that the investor has the potential to perform a certain task. Controllability refers to the external factors that an investor can control easily and perform well.

The concept of cognitive biases was first introduced by Kahneman and Tversky (1972) as errors in judgment, some of which are related to memory and others to the problem. Heuristic biases relate to mental shortcuts used in decision-making (Gutierrez et al., 2020) that often result in systematic errors in judgment (Kahneman and Tversky, 1972). In the stock exchange market, heuristic bias is a common phenomenon that affects investors (Tversky and Kahneman, 1974). Availability heuristic refers to the decision-making of investors based on how easy it is to bring something to mind, while representativeness heuristic helps the investors to compare the information with our mental prototypes (Rasheed et al., 2018).

Research has made valuable contributions to prospect theory and the literature on investment decision-making and cognitive biases by empirically showing that cognitive biases like heuristic thinking, overconfidence, anchoring, and confirmation biases significantly affect decision-making on investments (Hoffmann and Post, 2016). However, research on the intervening and moderating mechanisms of the relationship between the heuristic cognitive bias and decision-making is scarce, so why and when cognitive biases affect decision-making on investments remains unknown.

Drawing mainly on prospect theory, this study aims to fill in these gaps by examining the relationship between heuristic biases and investors’ irrational decision-making. However, considering the lack of research on the mediating and boundary conditions of this relationship, our primary goal is to explore and bring to the fore the intervening mechanisms (risk perception) and boundary conditions (extraversion) of the relationship (Kc, 2020). We build on prospect theory to propose that investors’ risk perceptions mediate the relationship, as risk perception is a vital component in the decision-making process. In fact, minimizing risk is investors’ key consideration in choosing an investment or initiating a project (Wood and Zaichkowsky, 2004). Risk perception refers to a subjective judgment that deals with individuals’ perception of the severity of a risk (Singh and Bhowal, 2010) when they evaluate uncertain activities (Slovic, 1987; Sartori and Ceschi, 2011). Investors’ beliefs, thoughts, and judgments shape their risk perceptions (Sachse et al., 2012; Nguyen et al., 2019), which affect their investment-related decision (Wood and Zaichkowsky, 2004). Since risk-taking is a common practice for investors (Hoffmann et al., 2015), cognitive biases alone cannot describe investors’ decision-making process; risk perception must be considered.

A common belief is that an investor keeps in mind the risk and return characteristics of a security or stock market while making financial decisions (Antonides and Van Der Sar, 1990; Ceschi et al., 2016; Li et al., 2020), but researchers have suggested that individuals’ decision-making is a complex combination of personality characteristics and demographics (Hallahan et al., 2003; Anbar and Eker, 2010; Young et al., 2012; Weller et al., 2018). We propose that individual personality characteristics like extraversion moderate the relationship between heuristic bias and risk perception and the relationship between cognitive biases and investors’ irrational decision-making (Holzmeister et al., 2020). Openness to experience, conscientiousness, extraversion, agreeableness, and neuroticism, also called the five-factor model, are commonly used personality traits (McCrae, 2004). This study focuses on extraversion because prior publications have shown that extraversion has a significant effect on financial decision-making (Oehler et al., 2018). Extraverted personalities tend to be involved in risky decision-making because they are more outgoing and optimistic, often paying high prices for financial assets (Sartori et al., 2017). Extraverts have dominant personalities and are bold in risk-taking (Nguyen et al., 2019). We also focus on extraversion as the most influential personality trait in financial and investment decisions (Costa and McCrae, 1980) because extraversion interacts with individuals’ emotional states, which influences individual investment behavior (McInish, 1980), risk perception, and decision-making (Alam et al., 2020). However, research has not explored the moderating effect of personality traits, including extraversion, in the relationship between heuristic cognitive bias and investors’ irrational decision-making.

**FIGURE 1:**
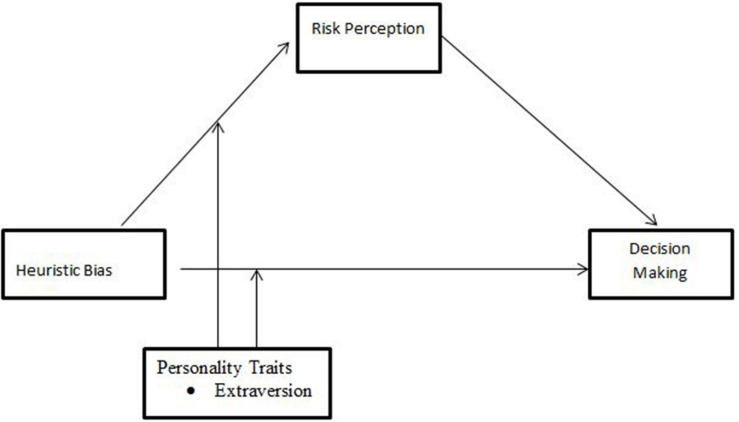
Conceptual framework.

Addressing this gap matters because of the variations in individuals’ cognitive biases that are due to their personality characteristics and the potential of these characteristics to influence their risk perceptions and irrational decision-making (Jurevièienë et al., 2020). This study addresses the moderating role of extraversion in both the direct and indirect relationships between heuristic biases and investors’ irrational decision-making. We suggest that individuals’ characteristics provide a way to understand the interrelationships among heuristic bias, risk perception, and investors’ irrational decision-making. The proposed model is presented in Figure 1.

In short, the objective of this study is to determine the effect of heuristic biases on irrational decision-making in the presence of risk perception and personality traits. The study finds that investors’ risk perceptions is a mechanism that underlies the relationship between cognitive bias (heuristic bias) and investors’ irrational decision-making, thus advancing our understanding of how heuristic biases affect irrational decision-making, both directly and indirectly, via risk perception. Investors’ thinking and emotions vary across personality traits, which can affect their perceptions of risk and their investment decisions, but there is little evidence about whether personality traits affect the relationship between heuristic biases and risk perception or the relationship between heuristic biases and investors’ irrational decision-making. We fill this void by testing the moderating effect of extraversion on the associations of heuristic biases with risk perception and irrational decision-making.

## Theory and Hypotheses Development

### Prospect Theory

Kahneman and Tversky’s (1979) prospect theory suggests that, when the outcome is uncertain, then the investor chooses to base their decision on a perceived opportunity for gain, rather than the perceived risk of loss (Ceschi et al., 2019).

In explaining investors’ decisions, many researchers have focused on cognitive biases and risk perception, given the traditional thinking that everyone is rational and uses all available information. However, behavioral finance paradigms highlight that individual thinking, emotions, and judgment errors are also reflected in investment decisions, rather than their being based only on market information. Investors’ behavior is not always rational because it is sometimes based on psychological and attitudinal motives.

### Cognitive Bias and Investment Decision-Making

In large, uncertain markets, complicated decisions are based on investors’ intuition, perceptions, emotions, and thinking (Kahneman and Riepe, 1998), but these decisions are often irrational, as cognitive biases are involved and complete information is ignored (Du and Budescu, 2018). Research has suggested that investors have cognitive biases in the form of mental shortcuts, like heuristics, and overestimate their abilities, expertise, and knowledge (Simon et al., 2000), thus often making decisions quickly.

Investors often face a shortage of time in making decisions in complex situations because variations in share prices occur within a few seconds, so they create heuristic biases to save time (Zaleskiewicz, 2006; Shah et al., 2019). Without time to make a sophisticated probability assessment, investors make decisions based on immediately available information and estimate other values using mental short cuts like heuristics (Oehler et al., 2018). Heuristic bias is comprised of availability and representative bias (Rasheed et al., 2018). Availability bias refers to a concept or thought that comes immediately to mind when an individual makes decisions, and representativeness heuristic bias occurs when the same objects or events confuses people’s thinking regarding the probability of an outcome. According to Tversky and Kahneman (1974), availability and representative heuristics are not limited to laypeople but are also used by experienced investors. When an investor makes an investment decision, he or she may assume that the current scenario is similar to a previous one and evaluate its prospects in the same way. Investors’ mindsets are affected by financial intermediaries, who have a significant influence in financial markets because of price fluctuations and available information (Healy and Palepu, 2001). Available information changes the investors’ preferences, and irrelevant information sometimes causes a human error and changes an investor’s thinking. To get a competitive edge, investors react quickly and make decisions using the available information, suggesting that mental short cuts shape irrationality and affect investment decisions (Bowers and Khorakian, 2014). Moreover, heuristic biases occur for both experienced and inexperienced investors (Elliot et al., 2018). Therefore, we developed the following hypothesis:

H1. There is a significant positive relationship between heuristic biases and investors’ irrational decision-making.

### Risk Perception as a Mediator

Several studies have revealed the effect of cognitive thinking on a decision’s outcome (Ishaque, 2020), but few studies have examined the relationship between biases (anchoring, heuristic thinking, disposition effect, and overconfidence) and risk perception (Sartori et al., 2014). Kahneman and Riepe (1998) found that investors make judgments about the probabilities that a particular outcome will occur and assign values to these results. Thus, norms, beliefs, and values are jointly measured in the construction of their preferences with risky options. Ritter (2003) suggested that such preferences may also create misrepresentations and identified factors that affect an individual’s risk perceptions, such as cognitive biases like overconfidence and heuristic bias. If investors are aware of the level of risk, they can manage the situation more effectively and can gain more profit than they can if they are not so aware (Nguyen et al., 2019). In addition, being risk-averse affects investors’ decision-making ability because they tend to perceive investments as high risk (Nguyen et al., 2019).

Risk perception refers to the subjective judgment of the intensity and severity of risk (Slovic, 1987). Investors make tradeoffs between risk level and profit margin when making investment decisions about securities, but they often follow a risk-averse approach (Kahneman and Tversky, 1979), making substantial investments in securities that give a higher return for the same risk level. The risk level also affects investment decisions in the capital market (Menkhoff et al., 2006).

When investors face identical situations and scenarios then some investors concluded that the situation is low risk, whereas other investors perceive at high risk. If risk perception influences decision making then it is important to investigate the antecedents/factors of risk perception (Nutt, 1993). Some researchers investigated that decision-making with a greater exposure to cognitive biases may reduce the risk perception (Palich and Bagby, 1995; Busenitz and Barney, 1997). Heuristic bias is subjective and may vary from individual to individual (Busenitz and Lau, 1996). Although biases help individuals cope with their cognitive limitations, they may result in less rational, less comprehensive decision-making (Barnes, 1984).

If, as argued by different researchers, cognitive biases directly influence risk perception, and risk perception directly influences an individual’s decisions, then cognitive biases indirectly affect the decision through their effect on risk perception. In other words, risk perception mediates the relationship between cognitive biases and decision-making.

In this study, risk perception is used as a mediator or intervening variable. A meditational analysis determines the intermediary process that leads from the transmitted variable to the criterion variable (Baron and Kenny, 1986; Muller et al., 2005).

H2. Risk perception mediates the relationship between heuristic bias and decision-making.

### The Moderating Role of Extraversion

Oehler et al. (2018) investigated the effect of extraversion and neuroticism on investment decision-making. They concluded that extraversion and neuroticism significantly affect decision-making in asset markets and that extraverted individuals tend to be excitable and to demonstrate risk-seeking behavior. Extraverted investors are often risk-takers who make massive investments in risky assets (Sartori et al., 2017).

Extraverted investors work on communications to build their relationships (Lee and Tsang, 2001). Belcher (2010) explored the effect of extraversion and neuroticism on fund managers and found that these traits significantly affect investors’ decisions. Moreover, compared to other kinds of investors, extraverted investors tend to be risk-takers who invest heavily in financial assets (Belcher, 2010). Extraverted individuals are optimistic and active, and their decisions tend to be productive (Dorn and Huberman, 2005). In this study, extraversion is a moderating variable that, as defined by Muller et al. (2005), strengthens or alters the direction between the predictor variable and the outcome variable.

*H3. Extraversion moderates the relationship between heuristic biases and risk perception*, such that the relationship is stronger when extraversion is high.

*H4. Extraversion moderates the relationship between heuristic biases and irrational decision-making*, such that the relationship is stronger when extraversion is high.

As hypothesis 3 states, extraversion moderates the relationship between heuristic bias and risk perception such that the relationship is strong when extraversion is high. However, extraversion can also strengthen the indirect relationship between heuristic biases and irrational decision-making.

*H5. Extraversion moderates the indirect relationship between heuristic biases and irrational decision-making*, such that the relationship is stronger when extraversion is high.

## Research Method

### Data Collection and Analysis

Time-lagged (three rounds, 3 months apart) survey data were collected from 247 individual investors. Three hundred investors were contacted using the convenience sampling technique. (According to Etikan et al. (2016), when there are constraints of time and availability and with the consent of participants, convenience sampling is preferred). The sample size was determined by following Pallant (2005), who suggested five to ten respondents as a sample size against each item. Of these 300 investors, 272 gave informed consent to participate. Sealed return envelopes were provided containing the promise of confidentiality, the survey questionnaire, and an information sheet that explained the research objectives.

Data for the predicting variable and the moderating variable were gathered in the first round, along with data on the respondents’ age, gender, education, and experience. Data on the mediator (risk perception) and the outcome variable (decision-making) were collected in the second and third phases/rounds, respectively. We received 268, 257, and 249 responses in the first, second, and third rounds of data collection, respectively. After the data were screened for negligence and missing values, 247 usable responses were used to test the hypothesized relationships between the variables. The net response rate was 82.33%.

Data were analyzed using structural equation modeling (SEM) in AMOS 25.0 and Hayes’ PROCESS macro for SPSS 25.0. Fifty-five percent of respondents had a master’s degree and 45% had an undergraduate degree. The average age and experience of the respondents were 45.61 years and 8.45 years, respectively. The purpose of using a time-lagged design was to reduce the common method variance (Podsakoff et al., 2003), so Herman’s single factor was also calculated to examine the data for common method variance (Hair et al., 2010). All items were constrained into a single factor that explained 23.24% of the total variance, well below the threshold of 50% (Hair et al., 2010), suggesting that common method bias was not an issue in our data.

Demographic variables are taken as a controlled variable like gender, age, experience, qualification, and income, etc. These are the factors that affect investment decisions, as Hassan Al-Tamimi and Anood Bin Kalli (2009) reported that decision-making and financial literacy are influenced by the demographic characteristics of the respondents.

### Measurement

The heuristic variable consists of availability and representative heuristic bias (Tversky and Kahneman, 1974). The availability heuristic scale consists of five items: two items adopted from Kudryavtsev et al. (2013), two items adopted from Luong and Ha (2011), and one item adopted from Waweru et al. (2008). (“I prefer to sell stocks on the days when the value of the stock market index decreases” is a sample item). The representative heuristic was measured using six items: three items adopted from Sarwar et al. (2014), two items adopted from Waweru et al. (2008), and one item adopted from Luong and Ha (2011). (“I consider the past performance of a stock before investing in it” is a sample item). Earlier research used only these items to measure the heuristic variable. This study used all these items (combined) to measure the heuristic variable. CFA is applied to check the validity of the scale. Rasheed et al. (2018) also used the same availability and representative heuristic scale. Tversky and Kahneman (1974) also reported that heuristics bias deals with the availability and representative heuristic.

Irrational decision-making was measured using Scott and Bruce’s (1995) scale. (“When making an investment, I trust my inner feelings and reactions” is a sample item). Initially, questionnaires were distributed to 100 investors consisting of five dimensions of personality traits. Results reported that 72% of respondents are of the extravert personality type. Based on pre-stage analysis, the extraversion personality trait is taken as a moderating variable. The scale developed and validated by John and Srivastava (1999) was used to measure extraversion (eight items). (“I see myself as someone who is talkative” is a sample item measuring extraversion).

Risk perception was measured by adapting four items from a 40-item scale developed and validated by Weber et al. (2002). While this scale consists of six dimensions–social, ethical, investment, health/safety, recreational, and gambling—we used only the four items for the investment dimension to measure investors’ risk perception. (“I invest 10% of my annual income in stock market shares” is a sample item). The complete measurement scale is given in **Appendix 1**.

## Results

### Means, Standard Deviation, and Correlations

Means, standard deviation (descriptive), and correlations (inferential) are presented in Table 1.

**TABLE 1:** Means and correlations.

Construct	Means	SD	1	2	3	4	5	6
1. Heuristic bias	3.87	1.15						
2. Risk perception	3.50	1.04	0.25**					
3. Decision making	3.75	1.18	0.30**	0.24**				
4. Extraversion	3.48	1.11	0.12*	0.05	–0.04			
5. Age	45.61	11.76	–0.03	–0.07	–0.04	0.06		
6. Experience	8.45	5.50	0.02	0.03	0.01	0.03	0.83**	
7. Education	1.45	3.36	0.03	–0.06	0.04	−0.22**	–0.08	–0.09

n = 247. *P < 0.05. **P < 0.01 level (2-tailed). SD, standard deviation. Education: 1 = undergrad; 2 = master’s degree.

### Measurement Model

Our measurement model consisted of heuristic biases (availability heuristic bias and representative heuristic bias), risk perception, irrational decision-making, and extraversion. The model was assessed by using confirmatory factor analysis (CFA). One item for availability heuristic bias (AH5) and one item for risk perception (RP4) did not load significantly and was dropped. The fit indices – χ^2^(267) = 535.87, χ^2^/df = 2.01, RMSEA = 0.07, CFI = 0.92, IFI = 0.92, and TLI = 0.92 – indicate that the measurement model has a good fit with the data.

Maximum shared variance (MSV), average shared variance (ASV), and average variance extracted (AVE) indicate that the measurement model has a good fit with the data. Table 2 shows that AVE > 0.50, ASV < MSV, and MSV and ASV < AVE for all the variables. Moreover, all of the constructs’ square root values of AVE are greater than their inter-construct correlations, so the scales demonstrate satisfactory levels of discriminant and convergent validity. Cronbach alpha (α) > 0.70 (Table 2) shows that the internal consistency of the items is also satisfactory, as the range is reported by Fornell and Larcker (1981) and Hair et al. (2013).

**TABLE 2:** Reliability and convergent validity and discriminant validities.

Construct	1	2	3	4	CR	AVE	MSV	ASV
1. Heuristic bias	**0.75**				0.93	0.56	0.12	0.07
2. Risk perception	0.30	**0.79**			0.83	0.63	0.09	0.06
3. Decision making	0.27	0.30	**0.78**		0.89	0.61	0.12	0.07
4. Extraversion	0.13	0.05	–0.03	**0.73**	0.89	0.54	0.02	0.01

*n = 247. AVE, average value extracted; MSV, maximum shared variance; ASV, average shared variance; CR, composite reliability. Bolded values on the diagonals of columns 2 to 5 are the square root values of AVE.*

### Structural Model – Direct and Mediation Results

We assessed the structural model in two steps. First, the examination of the direct association of heuristic biases with irrational decision-making was examined, and shows a significant positive relationship between heuristic biases and irrational decision-making (β = 0.28, *p* < 0.001), so hypothesis 1 is supported. In the second step, investors’ risk perception was included as a mediator in the relationship between heuristic biases and irrational decision-making. We used bootstrapping to assess the significance of this mediating role.

The results, shown in Table 3, reveal a significant positive indirect relationship between heuristic biases and irrational decision-making via risk perception (β = 0.07, 95% confidence interval did not overlap with zero). Moreover, when risk perception is included, the direct relationship between heuristic biases and irrational decision-making becomes insignificant, so hypothesis 2, that risk perception fully mediates the positive relationship between heuristic biases and irrational decision-making, is supported.

**TABLE 3:** Direct and indirect effects and 95% confidence intervals (model 2).

	β	Lower limit	Upper limit
**Standardized direct effects**			
Heuristic biases → Risk perception	0.30*	0.10	0.47
Heuristic biases → Decision making	0.18	–0.03	0.38
Risk perception → Decision making	0.24*	0.07	0.40
**Standardized indirect effects**			
Heuristic bias → Risk perception Decision making	0.07*	0.02	0.17

*Empirical 95% confidence interval does not overlap with zero. n = 247 (a sample of size 2,000 for bootstrapping).

### Moderation Results

**FIGURE 2:**
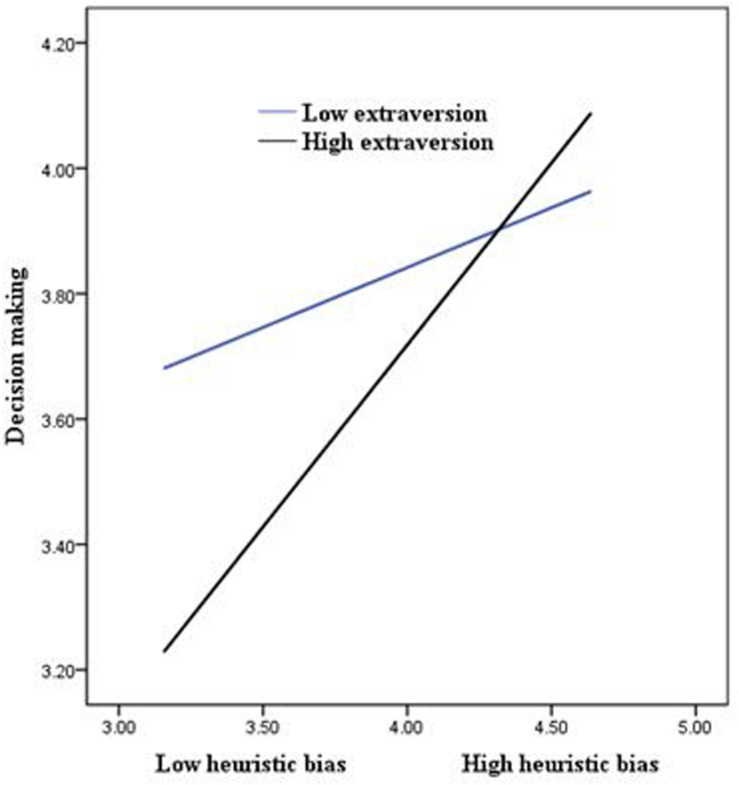
The moderating role of extraversion in the association between heuristic bias and decision making.

We used Hayes’ PROCESS model 8 to test the moderating effect of extraversion in the relationship between heuristic bias and risk perception (hypothesis 3), extraversion’s direct effect on the relationship between heuristic bias and irrational decision-making (hypothesis 4), and the moderated mediation, where extraversion moderates the indirect relationship (via risk perception) between heuristic bias and irrational decision-making (hypothesis 5). The results show that the effect of the interaction term between heuristic bias and extraversion on risk perception is significant (*B* = 0.30, *p* < 0.01), suggesting that extraversion moderates the positive relationship between heuristic bias and risk perception. These interactions were plotted at +1/−1 SD from the mean of extraversion (Figure 2). We ran a simple regression to examine the relationship between heuristic bias and risk perception at a low and high level of extraversion and found that the relationship is strong (*B* = 0.57, *p* < 0.001) when extraversion is high and also insignificant (*B* = 0.06, ns) when extraversion is low. Thus, hypothesis 3 is supported.

**FIGURE 3:**
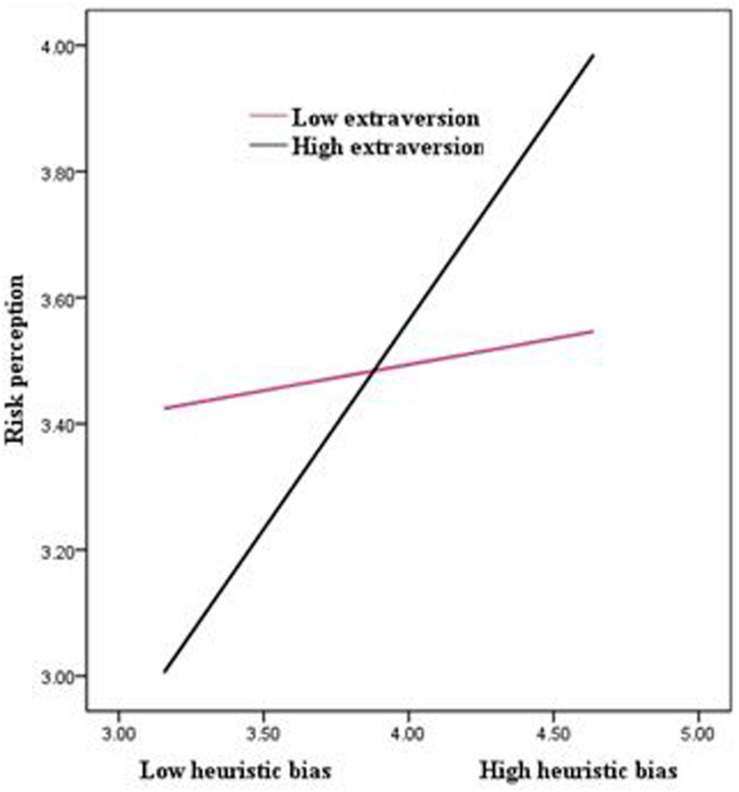
The moderating role of extraversion in the association between heuristic bias and risk perception.

The results also revealed that the effect of the interaction term between heuristic bias and extraversion on irrational decision-making is significant (*B* = 0.24, *p* < 0.01), suggesting that extraversion moderates the positive relationship between heuristic bias and irrational decision-making. These interactions were plotted at +1/−1 SD from the mean of extraversion (Figure 3). A simple slope test was conducted to examine the strength of the relationship between heuristic bias and irrational decision-making at high and low levels of extraversion. The results show that the relationship is significant (*B* = 0.47, *p* < 0.001) when extraversion is high and insignificant (*B* = 0.05, ns) when extraversion is low. Thus, hypothesis 4 is supported.

Finally, the results revealed that extraversion moderates the indirect relationship (via risk perception) between heuristic bias and irrational decision-making [bootstrap estimate = 0.05, bias-corrected CI (0.004, 0.11)]. As Table 4 shows, at high extraversion (+1 SD), the indirect relationship between heuristic bias and irrational decision-making is significant, but at low extraversion (−1 SD), the indirect relationship is insignificant. Thus, hypothesis 5 is supported.

**TABLE 4:** Extraversion as a moderator of the relationships of heuristic biases with risk perception and decision-making.

	Risk perception					Decision making				
	*B*	SE	*T*	LL	UL	*B*	SE	*T*	LL	UL
Age	–0.05	0.02	–2.88	–0.08	–0.01	–0.01	0.02	–0.58	–0.04	0.02
Experience	0.09	0.03	2.57	0.02	0.15	0.02	0.03	0.48	–0.05	0.08
Education	–0.11	0.12	–0.91	–0.37	0.14	0.07	0.13	0.60	–0.17	0.32
Heuristic biases	–0.72	0.32	–2.23	–1.34	–0.08	–0.57	0.31	–1.80	–1.19	0.05
Risk perception						0.16	0.06	2.53	0.03	0.28
Extraversion	–1.14	0.37	–3.07	–1.87	–0.41	–1.01	0.37	–2.74	–1.74	–0.28
Heuristic biases × extraversion	0.30	0.09	3.19	0.11	0.48	0.24	0.09	2.59	0.06	0.42
*R*^2^	0.12					0.14				

**Conditional indirect effect of heuristic bias on decision making via risk perception**										

			**Coefficient**		**Boot SE**	**LL (95% CI)**	**UL (95% CI)**			

Extraversion (−1 SD)			0.01		0.02	–0.02	0.06			
Extraversion (+1 SD)			0.09		0.04	0.01	0.19			

**Index of moderated mediation**										

			**Index**		**Boot SE**	**LL (95% CI)**	**UP (95% CI)**			

			0.05		0.02	0.004	0.11			

*N = 247, B, unstandardized regression coefficient. Bootstrap sample size = 5000. Confidence interval = 95%. LL, lower limit; UL, upper limit; SE, standard error.*

## Discussion – Theoretical Contributions and Practical Implications

We proposed that heuristic biases are positively related to investors’ irrational decision-making, both directly and indirectly, via investors’ risk perceptions, and that extraversion moderates both of these relationships. Based on survey data collected from 247 investors registered in various brokerage houses in Pakistan, the results of this study supported the hypothesized relationships.

The investors most likely to but those shares about which information is available instead of a complete analysis of the relevant information. The study support earlier literature about the notion that quick decisions have a significant effect on the effectiveness of investment decision-making (Hoffmann and Post, 2016; Du and Budescu, 2018) and suggest that investors use mental shortcuts in making their investment decisions. As an important contribution to the literature on investment decision-making, risk perception, and cognitive biases, the present study shows that investors’ risk perceptions significantly mediate the positive relationship between heuristic bias and irrational decision-making. Slovic (1987) investigated that risk perception is a subjective judgment caused by mental shortcuts and judgment errors (heuristic) which in turn affect decision-making. In this context, the results of this study also reported that risk perception mediates the relationship between heuristic bias and investment decisions. Given the scarcity of research on the intervening mechanisms of the relationship between cognitive biases and decision-making, this contribution is timely, relevant, and significant. It provides information on how the behavior of an investor reflects on the investor’s perception and decision-making. Our findings suggest that mental shortcuts, such as availability heuristics and representative heuristics, shape investors’ risk perceptions, which leads to their irrational behavior in making investment decisions. Thus, the present study extends our knowledge on how heuristic biases are related to investors’ irrational decision-making and the consequential potential of heuristic biases for shaping investors’ risk perceptions. By showing that investors’ risk perceptions mediate the positive relationship between heuristic biases and decision-making, the study also extends the nomological network of outcomes and antecedents of investors’ risk perceptions. The study also reveals that risk perceptions directly affect investment decisions and extends the literature by determining that better risk perceptions can improve decision-making (Nguyen et al., 2019).

The results of this study also reveal that extraversion moderates the direct and indirect relationships between heuristic biases and irrational decision-making. The findings suggest that extraverted investors demonstrate risk-seeking behavior, so they are more likely than introverted investors to invest in risky assets and use mental shortcuts in making their investment decisions. Thus, our findings contribute to the literature (Lee and Tsang, 2001; Belcher, 2010) that has suggested that individuals’ personality traits play an important role in their investment decisions. Our study highlights the moderating effect of the personality trait of extraversion, which strengthens the relationship between heuristic bias and decision-making.

By establishing extraversion as a moderator of the positive association between heuristic biases and irrational decision-making, the study contributes to the literature on the links between cognitive biases and investment decision-making and enhances the scope of personality traits in finding that extraversion strengthens the relationship between heuristic biases and investors’ risk perceptions.

The study’s findings could help investors, organizations’ policymakers, brokerage houses, and industrialists learn how risk perceptions influence their decision-making, how extraversion influences risk perceptions and decision-making, and that errors and deviations have significant effects on investors’ ability (thinking and reasoning) to make sound decisions. The study’s findings can help managers and policymakers understand the role of investors’ personality traits in their risk perceptions and decision-making and how cognitive biases vary based on personality traits. This study also contributes to prospect theory, as it explains how heuristic biases are linked to investors’ irrational decision-making through risk perceptions.

### Limitations and Recommendations for Research

Our study is based on data from Pakistani investors, so future research could study these relationships in other contexts to enhance the generalizability of our findings. We used a time-lagged design that reduced the common method variance, but drawing strong causal inferences may not be possible using the time-lagged design, so we suggest the use of experimental and longitudinal policies to draw causal inferences about the interrelationships among heuristic biases, investors’ risk perceptions, and investors’ irrational behavior in making investment decisions.

This study focuses on the heuristic bias, but many other cognitive biases, such as overconfidence, may affect investors’ decision-making and should also be considered. Moreover, we focused only on extraversion, so future research could examine the impact of other personality traits to enhance our understanding of the role of personality traits in investors’ irrational decision-making. Financial literacy may play a significant role as an independent variable in irrational decision-making in the presence of risk perception, so future research could also examine the effect of financial literacy on investors’ irrational decision-making. In addition, future research could look at the political impact of other biases on investors or perform a comparative study on the commodity market and equity market investors. To find the different behavioral effects, research could be performed on the individual investors of the stock exchange and commodity market investors. Moreover, demographic characteristics can be used as a moderating variable in future research.

## Data Availability Statement

The raw data supporting the conclusions of this article will be made available by the authors, without undue reservation.

## Ethics Statement

This study involving human participants was reviewed and approved by the Ethics Committee of the Department of Management Sciences, COMSATS University Islamabad, Lahore Campus, Lahore, Pakistan. The participants provided their written informed consent to participate in this study.

## Author Contributions

The authors identifies the research gap that how investors make a decision when there is a shortage of time and also how risk perception and extraversion personality shaping the behavior of investors. All authors contributed to the article and approved the submitted version.

## Conflict of Interest

The authors declare that the research was conducted in the absence of any commercial or financial relationships that could be construed as a potential conflict of interest.

## Figures and Tables

**TABLE A:** Measurement items.

Items	Description	References
	**Availability heuristic**	
AH1	I prefer to sell stocks on the days when the value of the stock market index decreases	Waweru et al. (2008);
AH2	I prefer to buy stocks on the days when the value of the stock market index increases	Luong and Ha (2011);
AH3	I prefer to invest in local stocks than international stocks because the information of local stocks is more available	Kudryavtsev et al. (2013)
AH4	I consider the information from my close friends and relatives as the reliable reference for my investment decisions	
AH5	I prefer to buy local stocks than trade in international ones	
	**Representative heuristic**	
RH1	I consider the past performance of the stocks before investing	Waweru et al. (2008);
RH2	I believe that through detailed analysis of past performance, future value of a contract in the stock market can be determined	Luong and Ha (2011);
RH3	I avoid investments in stocks that have a history of poor earnings	Sarwar et al. (2014)
RH4	I buy ‘hot’ stocks which provided the most return recently and avoid stocks that have performed poorly in the recent past	
RH5	I use trend analysis to make investment decisions	
RH6	Before investing I use trend analysis of some representative stocks to make investment decisions for all stocks	
	**Decision making**	
DM1	When making an investment, I trust my inner feelings and reactions	Scott and Bruce (1995)
DM2	I generally make investments that feel right to me	
DM3	When making investments, I rely upon my instincts	
DM4	When I make an investment, it is more important for me to feel the investment is right than have a rational reason for it	
DM5	When I make investments, I tend to rely on my intuition	
	**Personality traits**	
	**I see myself as someone who**	John and Srivastava (1999)
E1	Is talkative	
E2	Is reserved	
E3	Is full of energy	
E4	Generates a lot of enthusiasm	
E5	Tends to be quiet	
E6	Has an assertive personality	
E7	Is sometimes shy, inhibited	
E8	Is outgoing, sociable	
	**Risk perception**	
RP1	I invest 10% of my annual income in stock market shares	Weber et al. (2002)
RP2	I invest 5% of my annual income in a very speculative share	
RP3	I invest 5% of my annual income in a conservative stock	
RP4	I invest 10% of my annual income in government bonds (treasury bills)	
